# A challenge to human evolution—cognitive dissonance

**DOI:** 10.3389/fpsyg.2013.00179

**Published:** 2013-04-10

**Authors:** Leonid Perlovsky

**Affiliations:** The AFRL and Athinoula A. Martinos Center for Biomedical Imaging, Harvard UniversityCharlestown, MA, USA

## Cognitive dissonance challenges a possibility of human evolution

Cognitive dissonance (CD) is a discomfort caused by holding conflicting elements of knowledge. CD is among “the most influential and extensively studied theories in social psychology” (e.g., Alfnes et al., 2010, p. 147). It is well known that this discomfort is usually resolved by devaluing and discarding a conflicting piece of knowledge (Festinger, 1957; Cooper, 2007; Harmon-Jones et al., 2009); we discuss it in detail below. It is also known that awareness of CD is not necessary for actions to reduce the conflict, and these actions are often fast and momentary (Jarcho et al., 2011).

CD is particularly evident when a new scientific theory is developed. It takes a while to accept the new knowledge. However, I would like to emphasize that even a mundane element of knowledge to be useful it must differ from innate knowledge supplied by evolution or from existing knowledge acquired through experience. Otherwise the new knowledge would not be needed. For new knowledge to be useful it must contradict existing knowledge to some extent. Can new knowledge be complementary rather than contradicting? New knowledge does not come from nowhere, knowledge grows by analogy, by differentiation of previous knowledge, by using what already exists. This is the reason for several empirical laws: Zipf's law, the power law, Pareto laws. All of these laws essentially express equivalent statistical properties of systems, in which new entities (or usage) evolve from pre-existing ones (Simonton, 2000; Newman, 2005; Novak, 2010). For example, according to Zipf's law the frequency of a word is inversely proportional to its statistical rank; this empirical relation is observed in most languages and in many other similar systems (this was theoretically proven in the given references). Since new knowledge emerges by modifying previous knowledge, there must always be conflict between the two.

Because of this conflict between new and previous knowledge CD theory suggests that new knowledge should be discarded. This process of resolving CD by discarding contradictions is usually fast, “momentary” and according to CD theory new knowledge is discarded before its usefulness is established. This is the paradoxical conclusion of CD theory.

To summarize, according to CD theory knowledge has to be devalued and discarded. But accumulation of knowledge is the hallmark of human evolution. It follows that the fact of human cultural evolution contradicts this well established theory. This paradoxical aspect of CD has not received appropriate attention during more than 50 years of the development of CD theory.

## What has made human evolution possible?

The emergence of language accelerated the accumulation of knowledge. A powerful cognitive mechanism must have emerged in parallel with language, which would have enabled holding contradictory cognitions. A hypothesis advanced by Perlovsky (2006, 2010, 2012a,b, in press) suggests that music has been this powerful mechanism that enables us and our predecessors to maintain contradictory cognitions.

Motivations for this hypothesis are as follows. In non-human animals the vocal tract muscles are controlled from an old emotional center and voluntary control over vocalization is limited (Deacon, 1989; Larson, 1991; Davis et al., 1996; Schulz et al., 2005). Sounds of animal cries engage the entire psyche, rather than concepts and emotions separately.

Correspondingly, conceptual and emotional systems (understanding and evaluation) in non-human animals are less differentiated than in humans. When a monkey is scared by an approaching leopard, it does not think about what to say to the rest of the pack. In fact animals vocalize only when they are emotionally motivated. Every piece of conceptual knowledge is inextricably connected to the emotional evaluation of a situation, and to appropriate behavior, satisfying instinctual needs. The emotional and conceptual content is not differentiated, it is one undivided state.

Humans, in contrast, possess a remarkable degree of voluntary control over the voice, which is necessary for spoken language. In addition to the old mostly involuntary control over the vocal tract human have conscious voluntary control originating in the cortex. The evolution of language required this neural rewiring of circuits controlling vocalization. The human voice partly lost its dependence on uncontrollable emotions. Emotions in humans have separated from concepts and from behavior. The gradual differentiation of mental states with a significant degree of voluntary control over each part (emotions, concepts, behavior) gradually evolved along with language and brain rewiring. This differentiation destroyed the primordial unity of the psyche. With the evolution of language the human psyche lost its unity—the inborn connectedness of knowledge, emotions, and behavior.

The unity of psyche is paramount for concentrating the will and for survival. Those of our progenitors who could combine the advantages of differentiated language and knowledge with the unity of psyche and the ability to concentrate the will received survival benefits. The above considerations led to the following hypothesis: while part of the human voice evolved into language, acquired concrete semantics, and lost some of its emotionality, another part of the voice evolved into a less concretely semantic but powerfully emotional ability—toward music—helping to unify the split psyche. Of course these considerations are not “proofs.” This hypothesis has required experimental verifications.

## Review of experimental results

In one classical CD experiment (Aronson and Carlsmith, 1963) children devalued a toy if they were told that they couldn't play with it. This experiment has been reproduced thousands of times with both children and adults (Cooper, 2007) in various situations, confirming CD theory. The desire “to have” contradicts the inability “to attain”; this CD is resolved by discarding the contradiction. Aesop described this predicament 2500 years ago: the fox unable to attain the grape devalues the contradictory cognition by deciding that “the grape is sour.”

However, when the above experiment was reproduced with music playing in the background the toy was *not* devalued (Masataka and Perlovsky, 2012). Another experiment reproduced the so-called Mozart effect: student's academic test performance improved after listening to Mozart (although this was later “debunked,” any improvement was proven to be short-lived, Thompson et al., 2001). However, Perlovsky et al. (2013) used the Mozart effect to explore cognitive functions of music, this publication demonstrated (1) that students allocate *less* time to more difficult and stressful tests (as expected from CD theory), and (2) with music in the background students *can* tolerate stress, allocate *more* time to stressful tests, and improve grades.

These experiments tentatively confirmed the hypothesis that music helps overcome undesirable consequences of CD. It follows that music likely performs a fundamental cognitive function; music makes possible the accumulation of knowledge and thereby stimulates human evolution.

The origin, power, and evolution of our musical abilities were considered the “greatest mystery” by Darwin (1871), as well as a topic requiring explanation by Aristotle (1995). Unifying a psyche split by language, enabling the accumulation of knowledge and human evolution may all be part of the fundamental cognitive function of music—explaining music's origin and evolution from animal cries to Bach and Lady Gaga (Perlovsky, in press).

## Remaining unknowns and future research

Why have researchers of CD theory, “the most influential and extensively studied theory in social psychology” not noticed this contradiction between its fundamental premise and the fact of human evolution? This question by itself might be a topic of future research.

For people that are amusical, does it mean that they cannot participate in cultural evolution? How do they survive? A preliminary hypothesis is that they participate in cultural evolution by sharing conceptually, through language's cultural benefits, which have been initially accumulated with the help of musical ability. In principle this is no different from everyone sharing technological benefits created by scientists and engineers. Still this hypothesis requires scientific proofs; this is a wide field of future research. For example, preliminary data indicate that amusical students have lower grades (Perlovsky et al., 2013). Is this general academic deficiency a consequence of musical deficiency, or are both consequences of a more general condition?

Is CD an *emotional* discomfort? If so, which emotions? Consider this example: a young post-doc receives two offers, one from Stanford and one from Harvard. Each offer by itself would be a source of strong positive emotions (basic emotions of pride, etc.). But having to make the choice between the two could be stressful. This proves that the emotions of choice involving CD are different from well-studied basic emotions. How do we measure them (Bonniot-Cabanac et al., 2012)? How many different CD emotions exist? Does contradiction between any two cognitions elicit different CD emotions?

Are CD and musical emotions related? How do we measure musical emotions? How many of them exist? Why, during decades of studying emotions, have most efforts been concentrated on basic emotions that can be named by words (Izard, 1977)? Does the lack of a linguistic term for an emotion necessarily place the emotion outside the realm of scientific inquiry?
